# Ordinal regression models for zero-inflated and/or over-dispersed count data

**DOI:** 10.1038/s41598-019-39377-x

**Published:** 2019-02-28

**Authors:** Denis Valle, Kok Ben Toh, Gabriel Zorello Laporta, Qing Zhao

**Affiliations:** 1 School of Forest Resources and Conservation, University of Florida, Gainesville, Florida United States of America; 2 School of Natural Resources and Environment, University of Florida, Gainesville, Florida United States of America; 3 Setor de Pos-graduacao, Pesquisa e Inovacao, Faculdade de Medicina do ABC, Santo Andre, Sao Paulo Brazil; 4 Centro de Engenharia, Modelagem e Ciencias Sociais Aplicadas, Universidade Federal do ABC, Santo Andre, Sao Paulo Brazil

## Abstract

Count data commonly arise in natural sciences but adequately modeling these data is challenging due to zero-inflation and over-dispersion. While multiple parametric modeling approaches have been proposed, unfortunately there is no consensus regarding how to choose the best model. In this article, we propose a ordinal regression model (MN) as a default model for count data given that this model is shown to fit well data that arise from several types of discrete distributions. We extend this model to allow for automatic model selection (MN-MS) and show that the MN-MS model generates superior inference when compared to using the full model or more traditional model selection approaches. The MN-MS model is used to determine how human biting rate of mosquitoes, known to be able to transmit malaria, are influenced by environmental factors in the Peruvian Amazon. The MN-MS model had one of the best fit and out-of-sample predictive skill amongst all models. While *A. darlingi* is strongly associated with highly anthropized landscapes, all the other mosquito species had higher mean biting rates in landscapes with a lower fraction of exposed soil and urban area, revealing a striking shift in species composition. We believe that the MN and MN-MS models are valuable additions to the modelling toolkit employed by environmental modelers and quantitative ecologists.

## Introduction

Count data are ubiquitous in natural sciences^1–8^ and other fields^9–13^. The default modeling choice for count data has traditionally been a Poisson regression but it is widely acknowledged that a Poisson likelihood is a poor choice for over-dispersed and/or zero-inflated data and different conclusions may be reached depending on whether zero-inflation and/or over-dispersion are properly accommodated or not^3,8,14^. As a result, considerable research has been devoted to devising alternative statistical modeling approaches to properly accommodate these count data characteristics. A common alternative to the Poisson regression model that accounts for over-dispersion is the negative-binomial [NB] regression model^6,10,11,14,15^. However, other models also exist (e.g., new parameterization of the NB distribution that allows for different quadratic mean-variance relationships^7^, the Generalized Poisson distribution^12^, and the Quasi-Poisson regression^2^). Similarly, besides the negative-binomial regression model^1,16^, various hurdle and mixture models have been proposed in the literature to appropriately deal with zero-inflation (ZI)^3,4,8^.

As a result of the large number of potential models for count data and the fact that model choice has important consequences for the derived conclusions, choosing the most appropriate model is critical, even amongst models that properly accommodate over-dispersion and/or zero-inflation^2,3,7,8,14^. Despite substantial research comparing different statistical models using a range of criteria^1,3,12,16,17^, several researchers have ultimately concluded that determining the best modeling approach for count data is challenging^2,7^.

In this article, we propose a Bayesian ordinal regression model that can flexibly fit count data that arise from various distributions, regardless of zero-inflation and/or over-dispersion, circumventing the need to choose the most appropriate distribution. Furthermore, we extend this model to allow for model selection and parameter estimation within a single coherent modeling framework, enabling researchers to more fully explore the information from covariates (e.g., by accounting for non-linear relationships). We compare the performance of the proposed model to that of other commonly used models using simulations and real data. More specifically, our simulations explore how well the proposed model works for inferential purposes, including how well it (a) fits data that arise from different distributions, (b) determines which predictors are associated with the response variable (i.e., model selection), and (c) characterizes the (possibly nonlinear) relationship between the response variable and predictor variables. Our case study focuses on determining how land-use/land-cover and precipitation influence malaria risk by modeling mosquito data collected in the Peruvian Amazon. Finally, we end this article with a discussion on important topics for future research.

## Methods

### Basic model formulation (MN model)

A multinomial distribution can approximate any given discrete marginal distribution, with or without zero-inflation and/or over-dispersion. As a result, we rely on the multinomial distribution as the basis of our model and we hypothesize that an ordered multinomial probit model (MN model), also known as an ordinal regression model, can represent a wide range of regression models (i.e., conditional distributions).

Here we described the basic structure of a probit ordinal regression model^18^. We start by ranking the response variable *w*_*i*_ and let *y*_*i*_ = *rank* (*w*_*i*_), where ties are assigned the same ranking value (i.e., if *w*_*i*_ = *w*_*k*_, then *y*_*i*_ = *y*_*k*_ for *i* ≠ *k*). Therefore, *y*_*i*_ ∈ {1, 2, …, *J*} where J is the total number of unique *w*_*i*_ values. We assume that:

$$\begin{array}{c}{y}_{i}=1\,{\rm{if}}\,{z}_{i} < {b}_{1}\\ {y}_{i}=j\,{\rm{if}}\,{b}_{j-1} < {z}_{i} < {b}_{j}\,{\rm{for}}\,{\rm{j}}=2,\ldots ,{\rm{J}}-1\\ {y}_{i}=J\,{\rm{if}}\,{z}_{i} > {b}_{J-1}\end{array}$$

where *b*_1_, …, *b*_*J*−1_ are breaks to be estimated and *z*_*i*_ is a continuous latent variable. We further assume that *z*_*i*_ is given by:

$${z}_{i}\sim N({{\boldsymbol{x}}}_{{\boldsymbol{i}}}^{{\boldsymbol{T}}}{\boldsymbol{\beta }},1)$$

where ${{\boldsymbol{x}}}_{{\boldsymbol{i}}}^{{\boldsymbol{T}}}$ is the design vector and ***β*** is a vector of regression parameters. For identifiability purposes, we either have to set one of the breaks *b*_1_, …, *b*_*J*−1_ to zero or eliminate the intercept from our regression. We opt for the latter because it is not clear which break should be set to zero. Therefore, the design vector ***x***_***i***_ does not include a 1 for the intercept.

We use uninformative priors:

$$\begin{array}{c}[{b}_{1},\ldots ,{b}_{J-1}]\sim Unif(\,-\,100,100)I({b}_{1} < \ldots  < {b}_{J-1})\\ {\boldsymbol{\beta }}\sim N({\bf{0}},{\boldsymbol{I}})\end{array}$$

Finally, we note that the expected count is given by:

$$E[{w}_{i}|{{\boldsymbol{x}}}_{{\boldsymbol{i}}}]=\sum _{j=1}^{J}\,{u}_{j}\times p({y}_{i}=j|{{\boldsymbol{x}}}_{{\boldsymbol{i}}})=\sum _{j=1}^{J}\,{u}_{j}\times [{\rm{\Phi }}({b}_{j}-{x}_{i}^{T}\beta )-{\rm{\Phi }}({b}_{j-1}-{x}_{i}^{T}\beta )]$$

where Φ() is the cumulative density function of a standard normal distribution, *u*_*j*_ are the ordered unique values of *w*_*i*_, and *b*_0_ = −∞ and *b*_*J*_ = ∞. We rely on this expression for the expected count to create response curves depicting the effect of different covariates. The MN model can be fitted in a straight-forward fashion using standard methods in R, as illustrated in S1 Appendix.

### Simultaneously performing model fitting and model selection (MN-MS model)

The basic model formulation provided above can be extended to perform model selection and model fitting at the same time (MN-MS model). We start by noticing that the marginal probability associated with a particular model *M*_*k*_, defined by the subset of covariates k, can be calculated in closed form after integrating out the associated regression parameters ***β***_***k***_. This is given by:

$$\begin{array}{lll}p({M}_{k}|{\boldsymbol{z}}) & \propto  & \int N({\boldsymbol{z}}|{{\boldsymbol{X}}}_{k}{{\boldsymbol{\beta }}}_{{\boldsymbol{k}}},{\boldsymbol{I}}){\rm{N}}({{\boldsymbol{\beta }}}_{{\boldsymbol{k}}}|0,{\bf{I}})d{{\boldsymbol{\beta }}}_{{\boldsymbol{k}}}\\  & \propto  & exp\,(-\frac{1}{2}[-{{\boldsymbol{\mu }}}_{{\boldsymbol{k}}}^{{\boldsymbol{T}}}{{\boldsymbol{\Sigma }}}_{{\boldsymbol{k}}}^{-{\bf{1}}}\,{{\boldsymbol{\mu }}}_{{\boldsymbol{k}}}+{{\boldsymbol{z}}}^{{\boldsymbol{T}}}{\boldsymbol{z}}]){|{{\boldsymbol{\Sigma }}}_{{\boldsymbol{k}}}|}^{\frac{1}{2}}\end{array}$$

where $\{{{\boldsymbol{X}}}_{{\boldsymbol{k}}}^{{\boldsymbol{T}}}{{\boldsymbol{X}}}_{{\boldsymbol{k}}}+{\boldsymbol{I}}\}={{\boldsymbol{\Sigma }}}_{{\boldsymbol{k}}}^{-{\bf{1}}}$ and ${{\boldsymbol{\mu }}}_{{\boldsymbol{k}}}={{\boldsymbol{\Sigma }}}_{{\boldsymbol{k}}}{{\boldsymbol{X}}}_{{\boldsymbol{k}}}^{{\boldsymbol{T}}}{\boldsymbol{z}}$. In these equations, ***X***_***k***_ is the design matrix with only the subset of covariates k. Details on this integration can be found in S2 Appendix. Following Denison *et al*.^19^, we set the prior for each model *M*_*k*_ as $$p({M}_{k})\propto {(P+1)}^{-1}{(\begin{array}{c}P\\ {p}_{k}\end{array})}^{-1}$$, where *p*_*k*_ is the number of covariates in set k. In this prior, each number of covariates 0, …, *P* (P is the overall number of covariates) is assumed to be equally likely, represented by $\frac{1}{P+1}$. Furthermore, this prior assumes that all models with a given number of covariates *p*_*k*_ are equally likely, represented by $${(\begin{array}{c}P\\ {p}_{k}\end{array})}^{-1}$$, where $$(\begin{array}{c}P\\ {p}_{k}\end{array})$$ is the number of possible combinations of *p*_*k*_ elements out of P.

Our algorithm explores model space by randomly proposing the birth of a new covariate or the death or swap of an existing covariate. These proposed moves are then accepted or rejected using a standard Metropolis-Hastings acceptance ratio given by:

$${\rm{\min }}\{1,\frac{p({M}_{k}^{\ast }|{\boldsymbol{z}},\ldots )p({M}_{k}^{\ast })}{p({M}_{k}|{\boldsymbol{z}},\ldots )p({M}_{k})}\}=\,{\rm{\min }}\{1,\frac{exp\,(-\frac{1}{2}[\,-\,{{\boldsymbol{\mu }}}_{{{\boldsymbol{k}}}^{\ast }}^{{\boldsymbol{T}}}{{\boldsymbol{\Sigma }}}_{{{\boldsymbol{k}}}^{\ast }}^{-{\bf{1}}}{{\boldsymbol{\mu }}}_{{{\boldsymbol{k}}}^{\ast }}+{{\boldsymbol{z}}}^{{\boldsymbol{T}}}{\boldsymbol{z}}]){|{{\boldsymbol{\Sigma }}}_{{{\boldsymbol{k}}}^{\ast }}|}^{\frac{1}{2}}}{exp\,(-\frac{1}{2}[\,-\,{{\boldsymbol{\mu }}}_{{\boldsymbol{k}}}^{{\boldsymbol{T}}}{{\boldsymbol{\Sigma }}}_{{\boldsymbol{k}}}^{-{\bf{1}}}{{\boldsymbol{\mu }}}_{{\boldsymbol{k}}}+{{\boldsymbol{z}}}^{{\boldsymbol{T}}}{\boldsymbol{z}}]){|{{\boldsymbol{\Sigma }}}_{{\boldsymbol{k}}}|}^{\frac{1}{2}}}\times R\}$$

where *R* is typically equal to 1 and ${M}_{k}^{\ast }$ and *M*_*k*_ are the proposed and current models, respectively. This model selection procedure is done as part of the MCMC algorithm. A detailed description of this model formulation and associated algorithms can be found in Denison *et al*.^19^ and Zhao *et al*.^20^. We provide the derivation of the full conditional distributions used to create our Gibbs sampler in S2 Appendix. The implementation of our algorithm was done in R^21^. All the MN and MN-MS model results reported in this article are based on running our MCMC algorithm for 50,000 iterations and discarding the first half as burn in. The associated code, together with a short tutorial reproducing some of our results for the simulated data, is provided in S3 Appendix. Next, we describe our case study and the three sets of simulations that were performed to compare the performance of the proposed models in fitting data from different discrete distributions, identifying important predictor variables, and modeling nonlinear mean response functions.

### Simulation set 1: fitting different discrete distributions

To assess how well our ordinal regression model fits data from a variety of conditional distributions, with and without over-dispersion and/or zero-inflation, we generated 10 simulated datasets for each regression model (from a total of 12 distinct models; see distributional assumptions in Table 1). Each dataset contained 500 observations and the covariate x corresponded to 500 values equally spaced between −2 to 2. Parameter values were chosen to explore a range of possible scenarios. For instance, we simulated data with small and large means (*E*[*w*_*i*_|*x*_*i*_ = 0] = 1 and *E*[*w*_*i*_|*x*_*i*_ = 0] = 5, respectively). In addition to small and large means, we experimented with different combinations of small and large variances (n = 1 and n = 1/10, respectively) for the NB and ZINB models. In relation to zero-inflation, we assumed that the proportion of zeroes arising from the Bernoulli mixture component was equal to 0.25 when the covariate x was equal to zero (i.e., $p({q}_{i}=0|{x}_{i}=0)=\frac{1}{4}$).

**Table 1:** Assumptions used to simulated data for each model.

Reg. model	Mean	Variances	Assumptions	Parameter values
Poisson	Small	—	*w*_*i*_ ~ *Poisson* (*λ*_*i*_)	*β*_0_ = log (1); *β*_1_ = 0.5
	Large	—	*w*_*i*_ ~ *Poisson* (*λ*_*i*_)	*β*_0_ = log (5); *β*_1_ = 0.5
NB	Small	Small	*w*_*i*_ ~ *Neg Binom* (*μ*_*i*_ = *λ*_*i*_, *n*)	*β*_0_ = log (1); *β*_1_ = 0.5; *n* = 1
	Small	Large	*w*_*i*_ ~ *Neg Binom* (*μ*_*i*_ = *λ*_*i*_, *n*)	*β*_0_ = log (1); *β*_1_ = 0.5; *n* = 0.1
	Large	Small	*w*_*i*_ ~ *Neg Binom* (*μ*_*i*_ = *λ*_*i*_, *n*)	*β*_0_ = log (5); *β*_1_ = 0.5; *n* = 1
	Large	Large	*w*_*i*_ ~ *Neg Binom* (*μ*_*i*_ = *λ*_*i*_, *n*)	*β*_0_ = log (5); *β*_1_ = 0.5; *n* = 0.1
ZIP	Small	—	*q*_*i*_ ~ *Bernoulli* (*π*_*i*_) *w*_*i*_ ~ *Poisson* (*λ*_*i*_ × *q*_*i*_)	*α*_0_ = log (3); *α*_1_ = 0.5; ${\beta }_{0}=\,\mathrm{log}\,(\frac{4}{3});{\beta }_{1}=0.5$
	Large	—	*q*_*i*_ ~ *Bernoulli* (*π*_*i*_) *w*_*i*_ ~ *Poisson* (*λ*_*i*_ × *q*_*i*_)	*α*_0_ = log (3); *α*_1_ = 0.5; ${\beta }_{0}=\,\mathrm{log}\,(\frac{20}{3});{\beta }_{1}=0.5$
ZINB	Small	Small	*q*_*i*_ ~ *Bernoulli* (*π*_*i*_) *w*_*i*_ ~ *Neg Binom* (*μ*_*i*_ = *λ*_*i*_ × *q*_*i*_, *n*)	*α*_0_ = log (3); *α*_1_ = 0.5; ${\beta }_{0}=\,\mathrm{log}\,(\frac{4}{3});{\beta }_{1}=0.5;n=1$
	Small	Large	*q*_*i*_ ~ *Bernoulli* (*π*_*i*_) *w*_*i*_ ~ *Neg Binom* (*μ*_*i*_ = *λ*_*i*_ × *q*_*i*_, *n*)	*α*_0_ = log (3); *α*_1_ = 0.5; ${\beta }_{0}=\,\mathrm{log}\,(\frac{4}{3});{\beta }_{1}=0.5;n=0.1$
	Large	Small	*q*_*i*_ ~ *Bernoulli* (*π*_*i*_) *w*_*i*_ ~ *Neg Binom* (*μ*_*i*_ = *λ*_*i*_ × *q*_*i*_, *n*)	*α*_0_ = log (3); *α*_1_ = 0.5; ${\beta }_{0}=\,\mathrm{log}\,(\frac{20}{3});{\beta }_{1}=0.5;n=1$
	Large	Large	*q*_*i*_ ~ *Bernoulli* (*π*_*i*_) *w*_*i*_ ~ *Neg Binom* (*μ*_*i*_ = *λ*_*i*_ × *q*_*i*_, *n*)	*α*_0_ = log (3); *α*_1_ = 0.5; ${\beta }_{0}=\,\mathrm{log}\,(\frac{20}{3});{\beta }_{1}=0.5;n=0.1$

In these equations, *q*_*i*_ is a latent binary variable, ω_i_ is the response count variable, *x*_*i*_ is an explanatory variable, *λ*_*i*_ = exp (*β*_0_ + *β*_1_*x*_*i*_), and ${\pi }_{i}=\frac{\exp \,({\alpha }_{0}+{\alpha }_{1}{x}_{i})}{1+\exp \,({\alpha }_{0}+{\alpha }_{1}{x}_{i})}$. For the negative binomial distribution, *E*[*w*_*i*_] = *μ*_*i*_ and $Var[{w}_{i}]={\mu }_{i}+\frac{{\mu }_{i}^{2}}{n}$.

We fit our multinomial model with a quadratic specification (i.e., ${\beta }_{1}{x}_{i}+{\beta }_{2}{x}_{i}^{2}$) and compare model fit to that of models using the correct distributional assumptions. Because all models were fit under a Bayesian framework, we assess and compare model fit among these models using the posterior distribution of the log-likelihood (LLK), summarized by the median and 95% credible intervals (CI). Two models are judged to fit the data equally well if the 95% CI’s for their LLK overlap. If their 95% CI’s do not overlap, then the model with the highest LLK is judged to be the best fitting model. The models with the correct distributional assumptions (as described in Table 1) were fit using JAGS^22^. When using JAGS, the number of iterations was set to 10,000 and increased if necessary until all parameters had converged, as assessed by the potential scale reduction factor $\hat{R}$. Values of $\hat{R}$ smaller than 1.1 were assumed to indicate successful convergence.

### Simulation set 2: identifying relevant predictors

In our second set of simulations, we aim to examine if the multinomial model with model selection (MN-MS model) can adequately identify the few important predictor variables among a large number of covariates. To this end, we generated data from a Poisson regression model with a large number of covariates:

$${y}_{i}\sim Poisson\,(\exp \,({{\boldsymbol{x}}}_{{\boldsymbol{i}}}^{{\boldsymbol{T}}}{\boldsymbol{\beta }}))$$

where the design vector ${{\boldsymbol{x}}}_{{\boldsymbol{i}}}^{{\boldsymbol{T}}}$ contains the intercept, 10 covariates and all pairwise interaction terms between these 10 covariates. In total, this model has (1 + 10 + (10 × 9/2)) = 56 regression parameters in the vector ***β***. We simulate data by assuming that ***β*** is comprised of zeroes except for the intercept and a given number *m* (varying from 0 to 10) of randomly chosen elements of ***β***. These *m* non-zero elements in ***β*** were randomly set to 0.5 or to −0.5 and correspond to important predictor variables. We generated 10 datasets for each *m* = *0*, *1*, *2*, …, *10*, resulting in a total of 110 datasets with 500 observations per dataset.

According to a recent review, the most common procedure used for model selection in ecological publications is to select covariates based on AIC^23^, often within a forward, backward, or stepwise (i.e., combined forward and backward) approach. We compare the performance of this approach in identifying important predictors to that of the MN-MS model. To this end, we performed AIC model selection using the glm() and stepAIC() (from the MASS package) functions in R. The identified best model was subsequently fitted within a Bayesian framework. We compare the results from this best model to that of a Poisson model without any covariate selection and the MN-MS model. These latter models were also fitted within a Bayesian framework and we used the 95% credible intervals (CI) to determine if the method identified the zero and non-zero slope parameters correctly. More specifically, a non-zero coefficient was deemed correctly estimated if its 95% CI did not include zero and had the same sign as the true parameter. On the other hand, a zero coefficient was judged to be correctly estimated if the 95% CI overlapped with zero. Covariates that were excluded by the AIC model selection procedure were deemed to have a slope coefficient of zero.

### Simulation set 3: modeling nonlinear response curves

In this set of simulations, we investigate whether the multinomial model with model selection (MN-MS model) can approximate well different non-linear mean response functions in the absence of information on the correct distribution. To this end, we randomly generated 10 datasets, each of which had 500 observations with 6 predictor variables. We assumed that only the first 3 predictor variables influenced the mean response function, based on the following expression:

$${y}_{i}\sim NegBinom({\mu }_{{\rm{i}}}=\exp \,(\sin \,({x}_{1i})+3[\frac{\exp \,({x}_{2i})}{1+\exp \,({x}_{2i})}-0.5]+\,1-4{x}_{3i}+4{x}_{3i}^{2}),n=20)$$

where *E*[*y*_*i*_] = *μ*_*i*_ and $Var[{y}_{i}]={\mu }_{i}+\frac{{\mu }_{i}^{2}}{n}$. To approximate this mean response function, we rely on linear splines as our bases functions with four potential inflection points (i.e., knots) for each covariate, *a priori* set to 0.2, 0.4, 0.6, and 0.8 quantiles of the corresponding covariate.

### Case study: mosquito data from the Peruvian Amazon

Data on anopheline mosquitoes were collected along the Iquitos-Nauta road, in the Peruvian Amazon, between 2000 and 2001. The original study’s goal was to determine how different land-use land-cover (LULC) classes influenced malaria risk. To this end, Vittor *et al*.^9^ focused solely on *A*. *darlingi*, the mosquito species widely regarded as the most important malaria vector in the region, and performed a multinomial regression where biting rates were *a priori* classified as low, medium, or high. Overall, 56 sites (grouped into 14 spatial clusters) were sampled 15 to 16 times between 2000 and 2001. These data are fully described in Vittor *et al*.^9^ and a review of how malaria is related to LULC in the Amazon can be found in Tucker-Lima *et al*.^24^. Here we revisit this study but now using the proposed statistical method and using data on the six most common anopheline species in this dataset (i.e., *A*. *darlingi*, *A*. *nuneztovari*, *A*. *triannulatus*, *A*. *benarrochi*, *A*. *oswaldoi*, and *A*. *rangeli*), all of which are known to be able to transmit malaria in the region. We note that adequately modeling these data is challenging because the data are zero-inflated *and* over-dispersed (Table 2).

**Table 2:** Data on mosquito human biting rate is zero-inflated and over-dispersed.

Species	Proportion of zeroes	Maximum number of mosquitoes caught in a 6 hour period
*A*. *darlingi*	0.70	109
*A*. *nuneztovari*	0.92	24
*A*. *triannulatus*	0.60	308
*A*. *benarrochi*	0.82	249
*A*. *oswaldoi*	0.71	124
*A*. *rangeli*	0.86	33

The covariates in our model consist of precipitation, proportion of forest cover, and proportion of exposed soil/urban area. Precipitation data for each location and month were extracted from the Tropical Rainfall Measuring Mission (TRMM) product 3B43, which provides monthly rainfall estimates with a 0.25 × 0.25 degree spatial resolution^25^. LULC classification was based on a supervised random forest algorithm applied to a 2000 Landsat image with a 30 × 30 meter pixel, from which we calculated the proportion of terra-firme forest pixels and exposed soil/urban pixels within a buffer of 500 m around each point. All covariates were standardized to have a mean of zero and variance of one. Similar to the simulation study described above, we model potentially non-linear relationships through the use of linear spline bases, where knots were placed at 0.2, 0.4, 0.6, and 0.8 percentiles of each covariate.

We separately fit data from each of these six mosquito species using the MN and the MN-MS model. To determine how well these models fit and predict these data, we compare the log-likelihood (our measure of model fit) and out-of-sample predictive skill to that of a set of alternative models. As recommend by Roberts, *et al*.^26^, because we were primarily interested in spatial covariates (i.e., land use/land cover) and spatial predictions, out-of-sample predictive skill was determined through a spatial validation procedure. In this procedure, one spatial cluster of sites was removed for prediction purposes and the rest of the data were used to train the model in each of the 14 validation folds. Out-of-sample predictive performance was evaluated based on mean squared error (MSE). The alternative models were the Poisson, Negative-Binomial (NB), zero-inflated Negative Binomial (ZINB), and zero-inflated Poisson (ZIP) regression models, fitted with JAGS. All models in this comparison had the same set of covariates and spline terms.

## Results

### Simulation set 1: fitting different distributions

The MN model adequately accounted for over-dispersion and zero-inflation, having similar (based on overlapping 95% credible intervals) or greater goodness-of-fit when compared to that of the true models with estimated parameters (Table 3). The MN model only failed to fit well data originated from the ZIP model with large mean, with a worse fit in all ten simulated datasets. In this case, a comparison of the theoretical and the estimated distributions suggests that the MN model has difficulty representing conditional distributions that are approximately unimodal for small values of the covariate as well as strongly bimodal for large values of the covariate, with little probability mass for numbers in between both modes. Overall, these results highlight the flexibility of the MN model in adequately representing data generated from a wide range of distributions (over-dispersed and/or zero-inflated).

**Table 3:** The MN model fits well data generated from a diverse set of conditional distributions despite lack of information on the correct distribution.

Reg. model	Mean	Variances	MN model fits equally well or has better fit (proportion)
Poisson	Small	—	1.0
	Large	—	1.0
NB	Small	Small	0.9
	Small	Large	1.0
	Large	Small	1.0
	Large	Large	1.0
ZIP	Small	—	0.8
	Large	—	0.0
ZINB	Small	Small	0.9
	Small	Large	1.0
	Large	Small	1.0
	Large	Large	1.0

Numbers correspond to the proportion of datasets (based on 10 datasets) for which the MN model fitted the data equally well or had a better fit when compared to the true model with estimated parameters. Models were judged to fit the data equally well if their 95% credible intervals for the log-likelihood (our measure of goodness-of-fit) overlapped.

### Simulation set 2: identifying relevant predictors

**Figure 1:**
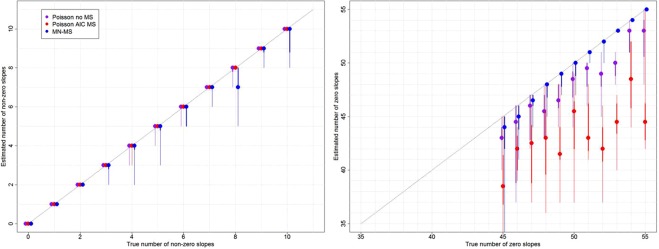
The MN-MS model performs slightly worse than the Poisson regression models in identifying the true non-zero slopes (left panel) but performs substantially better in identifying the true zero slopes (right panel). Results for the Poisson model without model selection (Poisson no MS; purple), with AIC model selection (Poisson AIC MS; red), and the MN model with model selection (MN-MS; blue) are displayed. A 1:1 line was added for reference (dashed diagonal line), where results closer to this line indicate better performance. Circles represent the median, thick lines represent the 20–80% range, while thin lines represent the full range (minimum to maximum) based on 10 datasets. Left panel: The x-axis displays the true number of non-zero slopes used to generate the data while the y-axis reveals how many of these slopes were correctly identified to be non-zero and were estimated with the correct sign. Right panel: The x-axis displays the true number of zero slopes used to generate the data while the y-axis reveals how many of these slopes were correctly identified to be zero.

Despite the MN-MS model performing slightly worse in identifying the relevant covariates than the Poisson regression model using all the covariates (“Poisson no MS”) and the AIC model selection procedure (“Poisson AIC MS”) (left panel in Fig. 1), the MN-MS model performed substantially better than the Poisson models in identifying the slopes that were equal to zero (right panel in Fig. 1). Indeed, the Poisson model using all the covariates (“Poisson no MS”) often times identified statistically significant slopes even when the corresponding covariates were independent of the response variable. Surprisingly, the AIC model selection method (“Poisson AIC MS”) was the worse approach in this respect, incorrectly identifying a relatively large proportion of “important” covariates. These results are striking because the Poisson models have the advantage of using the correct distributional assumption and yet the MN-MS model performs better overall.

### Simulation set 3: modeling nonlinear response curves

**Figure 2:**
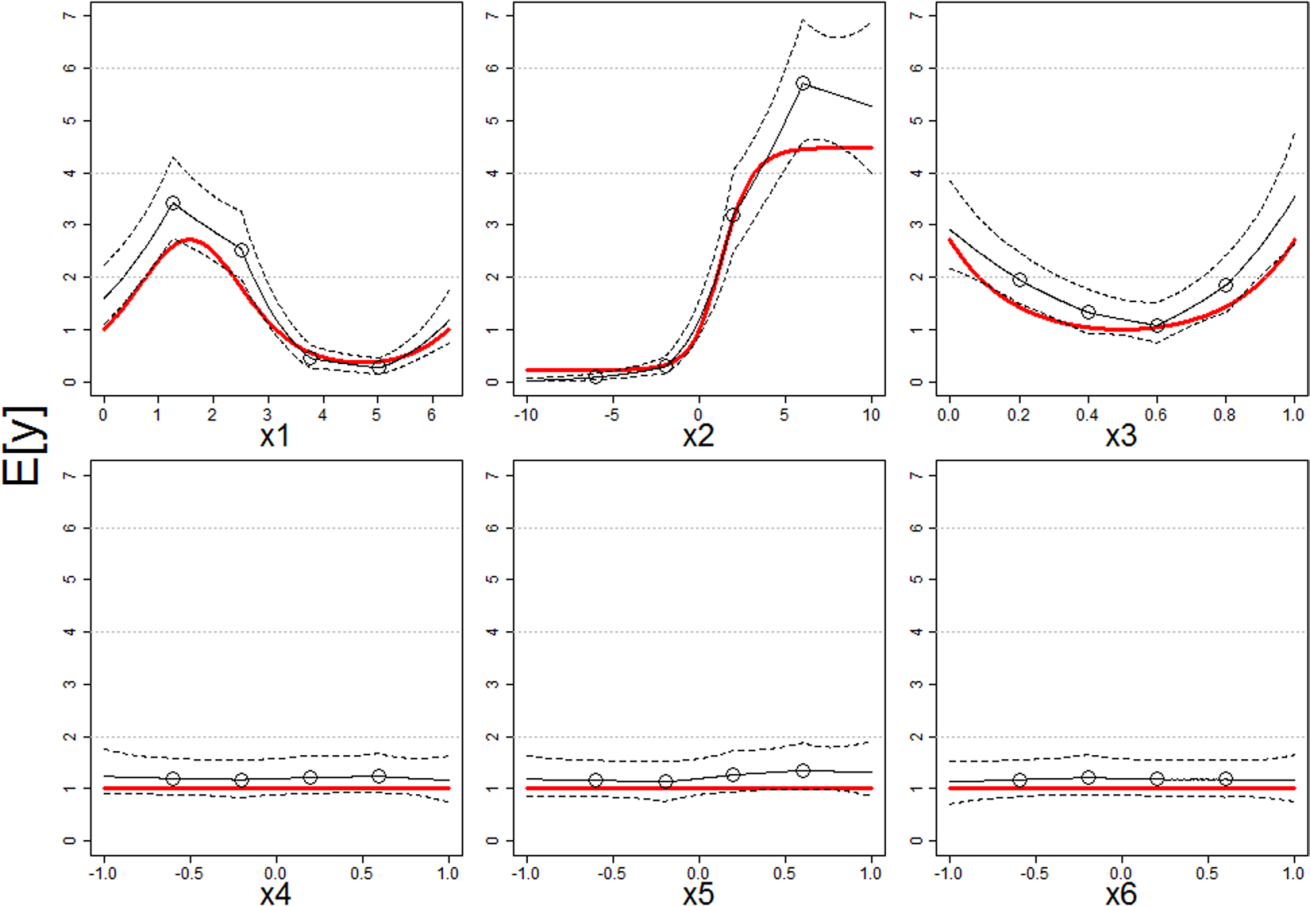
The MN-MS model estimates well non-linear effects of covariates *x*_1_, *x*_2_, and *x*_3_ (top panels) and the absence of effects associated with covariates *x*_4_, *x*_5_, and *x*_6_ (bottom panels). True mean response functions are depicted with red line while the estimated relationship are shown with black lines (continuous and dashed lines are the median and point-wise 95% credible intervals, respectively). Circles show the knot locations for each covariate, *a priori* set to 0.2, 0.4, 0.6, and 0.8 quantile of the corresponding covariate. The displayed response curves are based on one of the 10 simulated datasets and were created by only varying the focal covariate while the other covariates were set to their mean values.

We find that the MN-MS model can reliably estimate different non-linear relationships between covariates (e.g., sinusoidal, logistic, and quadratic functions for covariates *x*_1_, *x*_2_, and *x*_3_, respectively; top panels in Fig. 2) and the mean response using linear splines. Importantly, this model can also estimate well the absence of effects (e.g., covariates *x*_4_, *x*_5_, and *x*_6_; bottom panels in Fig. 2). These results suggest that the lack of information on the true distribution and the relationship between covariates and the mean response does not jeopardize the ability of the MN-MS model to infer these non-linear relationships. These results are important because researchers seldom have prior knowledge on the most appropriate distribution and mean response function to use to model their count data.

### Case study: mosquito data from the Peruvian Amazon

We find that the MN model was the best fitting model for five of the mosquito species and the second best model for the sixth remaining species (Table 4). The MN-MS model closely followed the model fit metrics of the MN model, being the best model for one mosquito species and the second best model for three other species. Overall, these results suggest that the MN and MN-MS models generally have superior fit to these zero-inflated and over-dispersed mosquito data when compared to other more standard regression models.

**Table 4:** The MN and MN-MS generally fit mosquito data better than other competing regression models.

Species	Model fit					
	Poisson	NB	ZINB	ZIP	MN	MN-MS
*A*. *darlingi*	−4756	−1283	−1245	−2682	−**1244**	−1245
*A*. *nuneztovari*	−407	−318	−311	−316	−**310**	−314
*A*. *triannulatus*	−6922	−1616	−1591	−3905	−**1551**	−1552
*A*. *benarrochi*	−4131	−775	−770	−1406	−**748**	−**748**
*A*. *oswaldoi*	−2533	−1057	−1035	−1682	−**1032**	−1033
*A*. *rangeli*	−1147	−587	−**539**	−670	−563	−568

The median of the log-likelihood (model fit) is provided for each combination of model and mosquito species. The best model for each species is emphasized in bold. “ZI” stands for zero-inflation.

The model fit statistics reported in Table 4 can be misleading for the identification of the best model if data are being over-fitted. Because models that over-fit the data have substantially worse out-of-sample predictive performance, we test if these models are over-fitting by comparing the models in Table 4 according to their out-of-sample predictive skill using a spatial block cross-validation procedure. This procedure reveals that both of the proposed models (MN and MN-MS models) tend to consistently have higher out-of-sample predictive skill (i.e., lower MSE values) than the other alternative models across all 6 mosquito species (Table 5). Interestingly, as shown in the right most column of Table 5, the MN-MS model tends to have a better predictive performance when compared to the MN model, with lower MSE for 4 mosquito species.

**Table 5:** The MN and MN-MS generally predict out-of-sample mosquito data better than other competing regression models.

Species	Predictive performance								
	MN model				MN-MS model				
	Poisson	NB	ZINB	ZIP	Poisson	NB	ZINB	ZIP	MN
*A*. *darlingi*	0.86	0.79	0.79	0.64	0.86	0.79	0.79	0.64	0.36
*A*. *nuneztovari*	0.86	0.86	0.93	1.00	0.93	0.79	0.93	1.00	0.57
*A*. *triannulatus*	0.79	0.71	0.79	0.64	0.79	0.71	0.79	0.64	0.29
*A*. *benarrochi*	0.79	0.79	0.86	0.71	0.79	0.86	0.93	0.71	0.79
*A*. *oswaldoi*	0.79	0.86	0.71	0.71	0.79	0.79	0.79	0.79	0.64
*A*. *rangeli*	0.79	0.93	0.93	0.79	0.71	0.93	0.93	0.79	0.79

Numbers indicate the proportion of cross-validation folds (based on 14 folds) in which the MN and MN-MS models had lower MSE scores when compared to each alternative model and for each mosquito species. “ZI” stands for zero-inflation. The last column on the right shows the proportion of cross-validation folds in which the MN-MS model had lower MSE score relative to the MN model.

**Figure 3:**
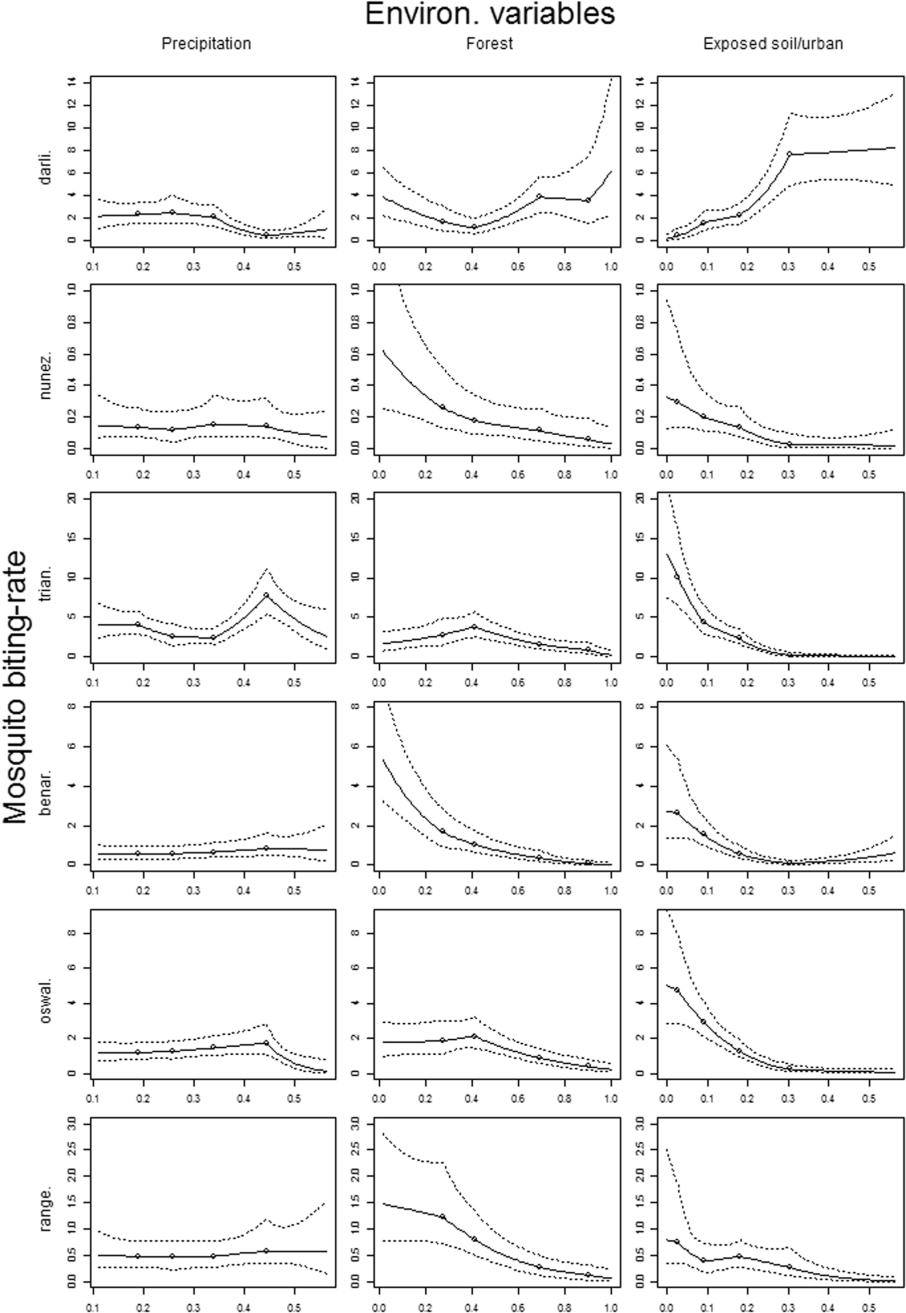
Statistical associations between mosquito biting-rates and environmental covariates based on the MN-MS model. Modeling results from individual mosquito species are shown separately in each row (*A*. *darlingi* = darli., *A*. *nuneztovari* = nunez., *A*. *triannulatus* = trian., *A*. *benarrochi* = benar., *A*. *oswaldoi* = oswal., and *A*. *rangeli* = range.). Continuous and dashed lines represent the median and the 95% credible intervals, respectively. Circles show potential inflection points (i.e., knot locations), *a priori* set to 0.2, 0.4, 0.6 and 0.8 quantiles of the covariate. Left to right panels show the inferred associations between mosquito biting-rate (number of mosquitoes caught per 6-hour period) and precipitation (mm/hr), proportion of forest pixels, and proportion of exposed soil/urban pixels, respectively. Proportion of pixels was calculated within a 500 m buffer of each observation location.

Using the MN-MS model, we find that the most important factors driving mosquito biting-rates were proportion of forest and exposed soil/urban area whereas precipitation had a comparatively minor role (Fig. 3). In general, we find a negative association between exposed soil/urban area and the biting-rate of all the mosquito species, except for *A*. *darlingi* which clearly is more common in more heavily disturbed areas (right panels in Fig. 3). Interestingly, three mosquito species (i.e., *A*. *nuneztovari*, *A*. *benarrochi*, and *A*. *rangeli*) also have higher biting-rates in areas with a lower proportion of forest (middle panels in Fig. 3), suggesting that these species thrive in areas that have some vegetation cover but that are not too pristine, such as secondary forest and agricultural lands. The use of linear splines allowed for the detection of several non-linear relationships in the mosquito data. For instance, Fig. 3 reveals that mosquito biting-rates for *A*. *rangeli* and *A*. *darlingi* tend to asymptote at intermediate levels of forest and exposed soil/urban area, respectively. Similarly, *A*. *triannulatus* and *A*. *oswaldoi* are only strongly influenced by precipitation within a specific range of this covariate.

**Figure 4:**
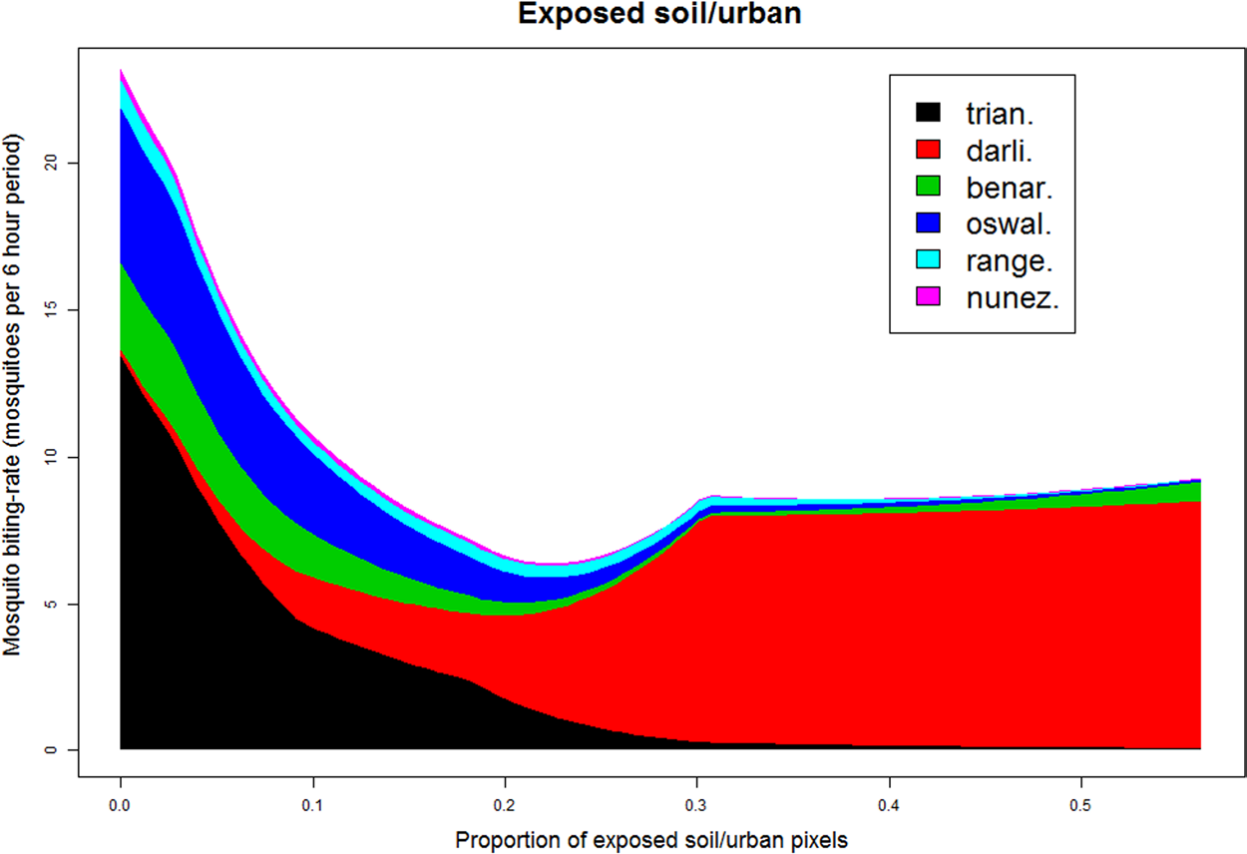
Large shift in species composition in mean mosquito biting-rates associated with changes in the proportion of exposed soil/urban area. Modeling results from individual mosquito species are shown in different colors (*A*. *darlingi* = darli., *A*. *nuneztovari* = nunez., *A*. *triannulatus* = trian., *A*. *benarrochi* = benar., *A*. *oswaldoi* = oswal., and *A*. *rangeli* = range.) as a function of the proportion of exposed soil/urban pixel. Proportion of pixels was calculated within a 500 m buffer of each observation location.

**Figure 5:**
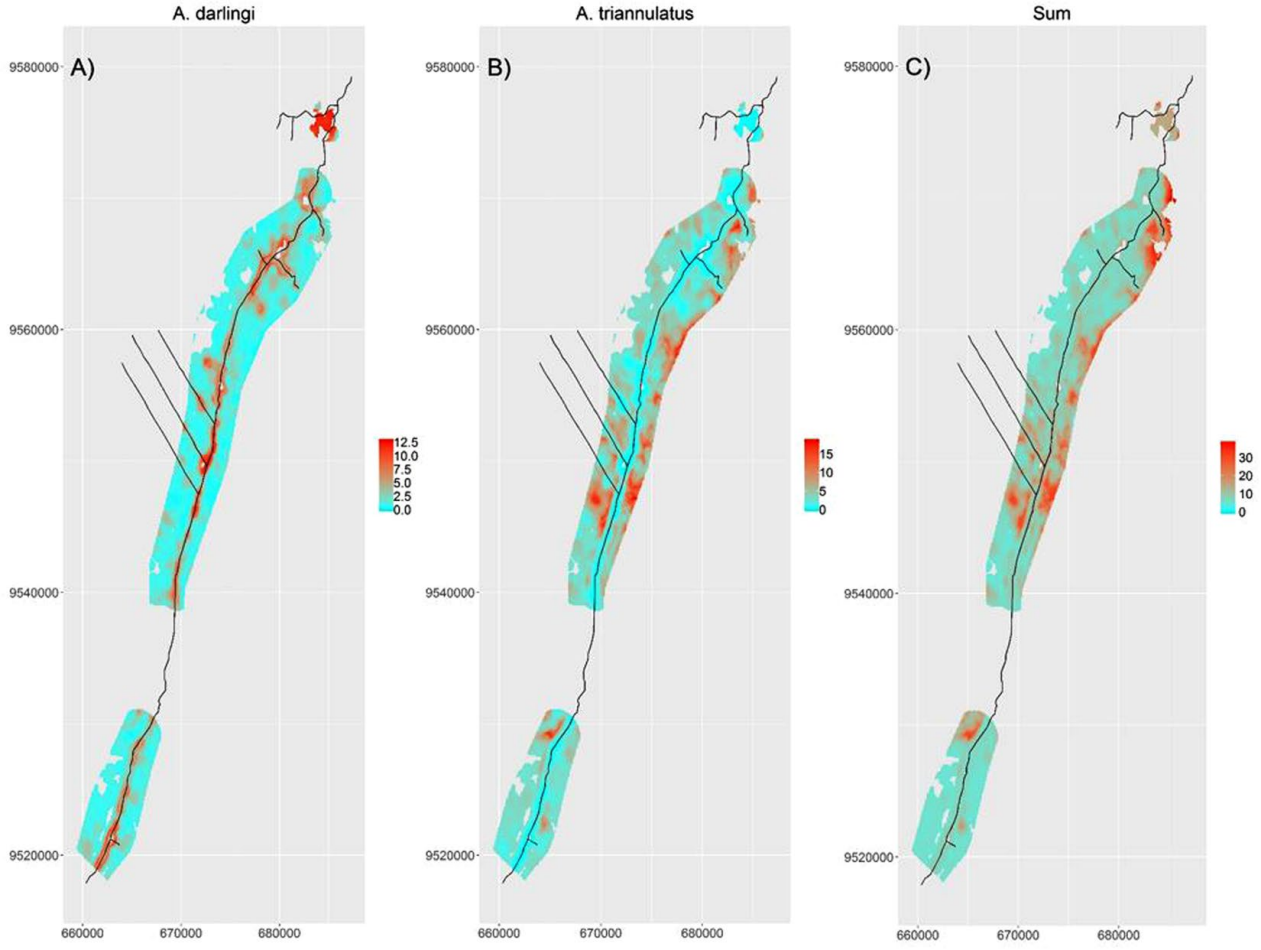
Spatial prediction of mean mosquito biting-rates for the two most common anopheline species and overall biting rate. From left to right, each panel shows the spatial prediction of mean mosquito biting-rate for *A*. *darlingi* (darli.), *A*. *triannulatus* (trian.), and the sum of the predicted mean biting-rate of the six anopheline mosquito species (Sum). Axes depict UTM coordinates in meters. The road network is depicted with black lines and covariate extrapolation is avoided by removing all areas for which covariate values were outside the range used to fit the model. Spatial extrapolation is avoided by restricting spatial prediction to within 2.5 km of sampled sites.

When results from individual species are put together, they reveal that areas with a lower proportion of exposed soil/urban pixels on average have a substantially higher overall mosquito biting-rate (Fig. 4). Interestingly, there is a pronounced shift in mosquito species composition as the proportion of exposed soil/urban area increases, with *A*. *darlingi* mosquitoes dominating areas with intermediate or high proportion of exposed soil/urban area. As expected, spatial predictions of mean mosquito-biting rate for *A*. *darlingi* reveals extremely high biting rates close to the primary road, reiterating the strong association of *A*. *darlingi* with highly anthropized sites (Fig. 5A). On the other hand, *A*. *triannulatus* (the other most common mosquito species in our sample) had a substantially different spatial pattern, being virtually absent from the immediate vicinity of the primary road (Fig. 5B), similar to the spatial pattern that emerges when the predicted mean biting rate for all 6 mosquito species are summed (Fig. 5C).

## Discussion

Count data are ubiquitous in multiple fields but these data are often zero-inflated and/or over-dispersed. There are several models that can be used to make inference based on data with these characteristics but determining the best one is challenging and often requires one to *a priori* choose a particular distribution. In this article, we have proposed a new statistical model that relies on a multinomial distribution to fit data from a wide range of different discrete distributions and automatically perform model selection. While ordinal regression models have a long tradition in statistics^18,27^, its use to flexibly model count data (rather than ordinal data) and perform model selection is, to our knowledge, a novel idea. We illustrate the features of our model using extensive simulations and apply this model to a case study on environmental drivers of malaria risk.

It is clear that the MN model can fit data from a wide range of conditional distributions, as evidenced by our simulation study. These simulation findings, together with one of the best model fit and out-of-sample predictive skill when applied to the mosquito data, suggest that the MN and MN/MN-MS models might be good default options for drawing inference from count data. While the data generated from the ZIP model with large mean was not well fit, had we chosen the wrong model for these data (i.e., a NB regression model, as suggested by^1^), the fit to these data would be substantially worse than that for the MN model (results not shown). Additional research will be needed to more precisely determine the conditions under which the MN model is likely to fail to fit well and how prevalent these conditions are.

Despite having no prior knowledge of the underlying distribution of the data, the MN-MS model performed very well in variable selection. While the MN-MS model was slightly worse in identifying true explanatory variables, this was greatly outweighed by its superiority in eliminating false predictors, resulting in overall better inference when compared to using a simple Poisson regression with or without AIC model selection. This improved performance is supported by other studies that have compared Bayesian model averaging with simple and stepwise regression methods^20,28^. Finally, our simulation results suggest that the adopted linear spline approach was able to capture a wide range of non-linear patterns. We chose linear splines because they are simple and straight-forward to implement but there is a wide-range of more flexible spline functions that could have been used (e.g., cubic splines, b-splines, and thin-plate splines)^29^. Regardless of the specific type, all spline approaches entail the inclusion of numerous additional “covariates” (i.e., basis functions) into the design matrix, a setting in which our model selection procedure is likely to be particularly effective (e.g.^30^).

In relation to our case study, we build on the original work of Vittor *et al*.^9^ in two important aspects. First, we examine multiple malaria vector species rather than just *A*. *darlingi*. This is important because, despite *A*. *darlingi* being widely acknowledged to be the main malaria vector in the Amazon region^31^, several other anopheline species have been shown to be competent vectors and to be locally important for malaria transmission^32–40^. The second aspect that was improved refers to the statistical modeling approach. Vittor *et al*.^9^ relied on a multinomial regression model where biting rates were classified as “low” (0–0.09/hr), “medium” (0.1–0.9/hr) and “high” (1.0–3.8/hr). We have improved on this modeling approach by avoiding the arbitrariness associated with data discretization, and the resulting loss of information, and by allowing for non-linear associations.

Our results suggest that one might arrive at very different conclusions regarding how land-use/land cover (LULC) classes are associated with malaria risk depending on which anopheline species is analyzed. Unlike the other mosquito species, *A*. *darlingi* seem to thrive in highly anthropized areas, greatly corroborating earlier published results^9,32,41^. Indeed, our model predicts that biting rate for this species concentrates close to roads, particularly in areas with a high proportion of exposed soil/urban area. However, the addition of other mosquito species reveals a different picture in that over-all biting rate is actually higher in areas with lower proportion of exposed soil/urban area. This is partly a result of significant changes in species composition along the urbanity gradient, where *A*. *triannulatus* dominates areas with less exposed soil/urban area whereas *A*. *darlingi* is the dominant species at the other side of the spectrum.

We believe that the proposed method will find wide use in natural sciences because it can flexibly fit and predict data with or without zero-inflation and/or over-dispersion while simultaneously identifying the most relevant explanatory variables.

## Supplementary information

Appendices S1, S2, and S3

Vittor et al 2006 data
