# Supplementary material for: Ordinal regression models for zero-inflated and/or over-dispersed count data
Source: Sci Rep. 2019 Feb 28;9:3046. doi: 10.1038/s41598-019-39377-x (PMC6395857; doi:10.1038/s41598-019-39377-x)
Supplement: Supplementary file 1 — Appendices S1, S2, and S3 [file 41598_2019_39377_MOESM1_ESM.pdf]

S1 Appendix. Brief tutorial on fitting the MN model using existing tools in R

Denis Valle

January 2019

Part of the article: Ordinal regression models for zero-inflated and/or over-dispersed count data.
Authors: Denis Valle, Kok Ben Toh, Gabriel Zorello Laporta, Qing Zhao

In this tutorial, we illustrate:

1) how to fit the MN model using existing tools in R within a frequentist approach. While the method described here does not allow for model selection, it does illustrate how simple it is to fit the proposed model to count data.
2) how the MN model can fit the data as well as the model with the true likehood
3) how the mean function is calculated for the MN model

Simulating data

We assume that there are 500 observations and a single covariate and generate data from a negative binomial distribution with n=20. The mean is assumed to be a quadratic function of the covariate (red line in the figure below).

```
rm(list=ls(all=TRUE))
set.seed(123)
nobs=500

#mean function
x1=seq(from=0,to=pi,length.out=nobs)
b0=1
b1=1
b2=-0.5
mean1=exp(b0+b1*x1+b2*(x1^2))

#generate response
y=rnbinom(nobs,mu=mean1,size=20)

#visualize generated data
plot(x1,y,xlab='Covariate',ylab='Counts')
lines(x1,mean1,col='red',lwd=3) #mean function
```

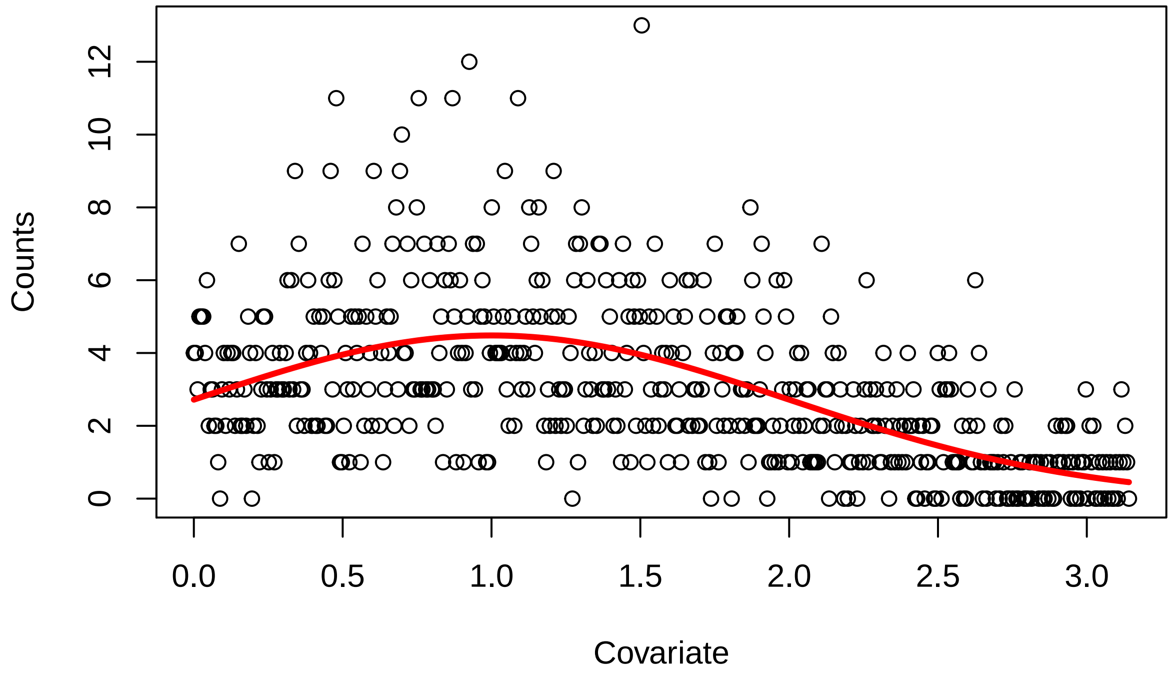


```
#output results
fim=data.frame(y=y,x1=x1)
setwd('U:\\modeling abundance\\appendix tutorial polr')
write.csv(fim,'fake data.csv',row.names=F)
```

Fitting the model

We fit the MN model through the use of the *polr()* function (*MASS* package) by converting the response variable into an ordered factor.

```
library('MASS')
set.seed(123)

#get data
setwd('U:\\modeling abundance\\appendix tutorial polr')
dat=read.csv('fake data.csv',as.is=T)
nobs=nrow(dat)
dat$x1sq=dat$x1^2
uni.y=sort(unique(dat$y))
dat$y1=factor(dat$y,ordered=T,levels=uni.y)

#fit model using a quadratic relationship for the mean
m <- polr(y1 ~ x1+x1sq, data = dat, Hess=TRUE,method='probit')
m1=summary(m)
```

```
#get estimated coefficients
coef1=m1$coef
ind=which(rownames(coef1)%in%c('x1','x1sq'))
slopes=coef1[ind,'Value'] #estimated slope coefficients
breaks1=c(-Inf,coef1[-ind,'Value'],Inf) #estimated break points
```

Then, we compare the fit of the MN model to that of a negative-binomial regression, the model with the true likelihood, fitted using the *glm.nb()* function. This comparison reveals that the MN model had a better fit to these data than the negative-binomial regression.

```
#log-likelihood for the MN regression model
-m$deviance/2
```

```
## [1] -943.901
```

```
#fitting a negative-binomial regression
m1 <- glm.nb(y ~ x1 + x1sq, data = dat)
tmp=summary(m1)

#log-likelihood for the negative-binomial regression
tmp$twologlik/2
```

```
## [1] -945.8729
```

Estimating the mean function and comparing it to the true mean function

We illustrate how the mean function is determined using the code below to calculate the expected value of y for different values of the covariate (i.e., $E[y|x]$). The comparison of the estimated and the true mean function reveals that the MN regression model estimates well the true mean function.

```
#calculate expected value of y for different values of x1 (i.e., E[y|x])
mean1=coef1[1]*dat$x1+coef1[2]*dat$x1sq
ymean=rep(NA,nobs)
for (i in 1:nobs){
  #get probabilities associated with the different y values
  prob=pnorm(breaks1[-1]-mean1[i])-pnorm(breaks1[-length(breaks1)]-mean1[i])
  #calculate the expected value
  ymean[i]=sum(prob*uni.y)
}

#plot results
plot(dat$x1,ymean,type='l',xlab='covariate',ylab='E[y]',ylim=c(0,5))

#compare to true relationship
b0=1
b1=1
b2=-0.5
true.mean1=exp(b0+b1*dat$x1+b2*(dat$x1^2))

lines(dat$x1,true.mean1,col='red')

legend(2.5,5,lty=1,col=c('black','red'),c('estim.','true'))
```

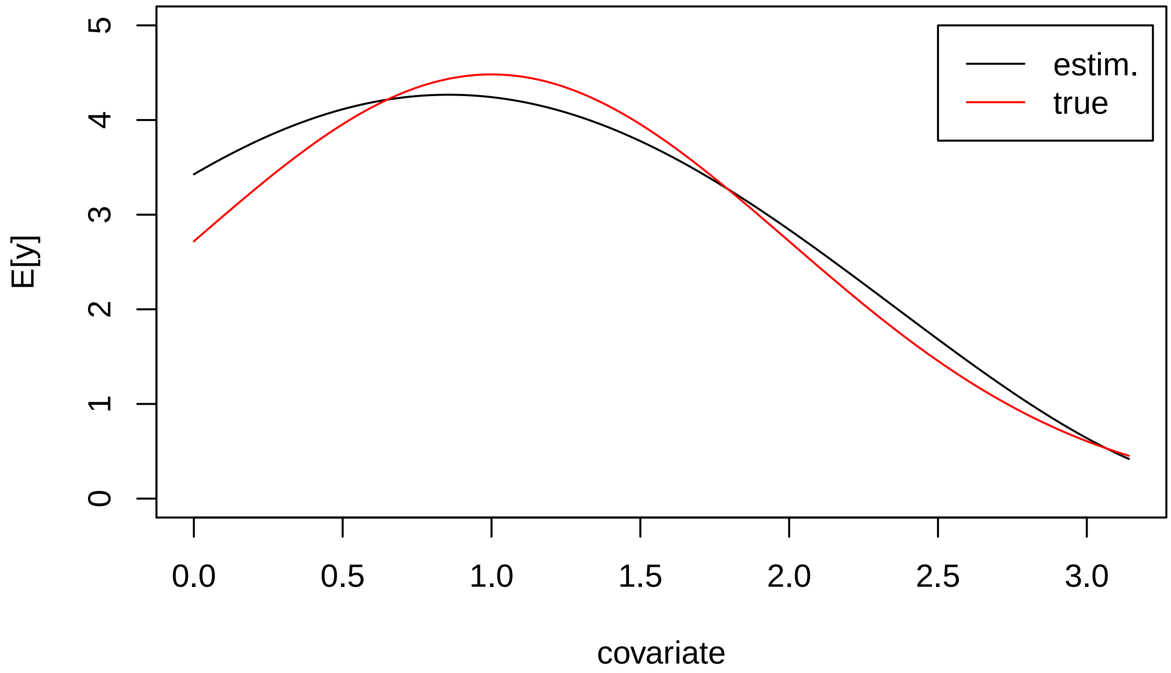
estim.
true
E[y]
covariate
0
1
2
3
4
5
0.0
0.5
1.0
1.5
2.0
2.5
3.0

S2 Appendix. Model description and full conditional distributions

Part of the article: Ordinal regression models for zero-inflated and/or over-dispersed count data

Authors: Denis Valle, Kok Ben Toh, Gabriel Zorello Laporta, Qing Zhao

1. *Statistical model*

Recall that, in our multinomial model, we assume that

$y_i = 1$ if $z_i < b_1$

$y_i = j$ if $b_{j-1} < z_i < b_j$ for j=2,…,J-1

$y_i = J$ if $z_i > b_{J-1}$

where $b_1, \ldots, b_{J-1}$ are breaks to be estimated and z_i is a continuous latent variable, given by:

$$z_i \sim N\left(x_i^T \beta, 1\right)$$

Furthermore, recall that our priors are given by:

$$\beta \sim N(0, I)$$

$$\left[b_1, \ldots, b_{J-1}\right] \sim Unif(0,100) I\left(b_1 < \cdots < b_{j-1}\right)$$

2. *Full conditional distributions (FCDs)*

To create our Gibbs sampler, we need to derive the FCD’s for each set of parameters in our statistical model. These FCD’s are provided below:

- For M_j:

To move between models with different covariates, we need to first integrate out the regression parameters β_j. Let the corresponding model be denoted by M_j. This integration is given by:

$$p\left(M_j | \ldots\right) \propto \int p\left(z | X_j M_j, I\right) p\left(\beta_j | 0, T_j\right) d\beta_j$$

$$\propto \int N\left(z | X_j \beta_j, I\right) N\left(\beta_j | 0, T_j\right) d\beta_j$$

$$= |2\pi I|^{-\frac{1}{2}} \left|2\pi T_j\right|^{-\frac{1}{2}} \int \exp\left(-\frac{1}{2}\left(z - X_j \beta_j\right)^T \left(z - X_j \beta_j\right)\right) \exp\left(-\frac{1}{2} \beta_j^T T_j^{-1} \beta_j\right) d\beta_j$$

$$= |2\pi I|^{-\frac{1}{2}} |2\pi T_j|^{-\frac{1}{2}} \int \exp\left(-\frac{1}{2}\left[\beta_j^T\{X_j^T X_j + T_j^{-1}\}\beta_j - 2\beta_j^T X_j^T z + z^T z\right]\right) d\beta_j$$

Let $\{X_j^T X_j + T_j^{-1}\} = \Sigma_j^{-1}$ and $\Sigma_j^{-1}\mu_j = X_j^T z$. Then:

$$= |2\pi I|^{-\frac{1}{2}} |2\pi T_j|^{-\frac{1}{2}} \int \exp\left(-\frac{1}{2}\left[\beta_j^T \Sigma_j^{-1}\beta_j - 2\beta_j^T \Sigma_j^{-1}\mu_j + \mu_j^T \Sigma_j^{-1}\mu_j - \mu_j^T \Sigma_j^{-1}\mu_j + z^T z\right]\right) d\beta_j$$

$$= |2\pi I|^{-\frac{1}{2}} |2\pi T_j|^{-\frac{1}{2}} \exp\left(-\frac{1}{2}\left[-\mu_j^T \Sigma_j^{-1}\mu_j + z^T z\right]\right) \times \int \exp\left(-\frac{1}{2}\left[\beta_j^T \Sigma_j^{-1}\beta_j - 2\beta_j^T \Sigma_j^{-1}\mu_j + \mu_j^T \Sigma_j^{-1}\mu_j\right]\right) d\beta_j$$

$$= |2\pi I|^{-\frac{1}{2}} |2\pi T_j|^{-\frac{1}{2}} \exp\left(-\frac{1}{2}\left[-\mu_j^T \Sigma_j^{-1}\mu_j + z^T z\right]\right) |2\pi \Sigma_\mathrm{j}|^{\frac{1}{2}}$$

$$\propto \exp\left(-\frac{1}{2}\left[-\mu_j^T \Sigma_j^{-1}\mu_j + z^T z\right]\right) |T_j|^{-\frac{1}{2}} |\Sigma_\mathrm{j}|^{\frac{1}{2}}$$

If we assume that $T_j = I_j$, as in the main manuscript, then $|T_j|^{-\frac{1}{2}}$ can be dropped from this expression, leading to:

$$\propto \exp\left(-\frac{1}{2}\left[-\mu_j^T \Sigma_j^{-1}\mu_j + z^T z\right]\right) |\Sigma_\mathrm{j}|^{\frac{1}{2}}$$

This expression is the main result that we use in the Metropolis-Hastings acceptance ratio when exploring model space by accepting/rejecting birth, death or swap moves.

- For z_i:

If $y_i = j$, then

$$p(z_i| \ldots) \propto N(z_i | x_i^T \beta, 1) I(b_{j-1} < z_i < b_j)$$

In other words, we sample z_i from a truncated normal distribution where the lower and upper truncation limits are given by the estimated breaks b_{j-1} and b_j, respectively.

- For β:

Regression parameters β can be sampled in closed form using standard conjugate results involving a normal likelihood and a normal prior.

$$p(\beta| \ldots) \propto N(z|X\beta, I) N(\beta|0, T)$$

$$\propto \exp\left(-\frac{1}{2}(z - X\beta)^T(z - X\beta)\right) \exp\left(-\frac{1}{2}\beta^T T^{-1}\beta\right)$$

$$\propto \exp\left(-\frac{1}{2}\left(-2\beta^T X^T z + \beta^T X^T X\beta + \frac{1}{10}\beta^T T^{-1}\beta\right)\right)$$

$$\propto \exp\left(-\frac{1}{2}(\beta^T[X^TX + T^{-1}]\beta - 2\beta^T X^T z)\right)$$

$$p(\beta| \dots) = N([X^TX + T^{-1}]^{-1}X^Tz, [X^TX + T^{-1}]^{-1})$$

- For b_j:

The breaks b_j specifies how the latent continuous variable z_i relates to the discrete y_i outcomes. These breaks are sampled from a uniform distribution:

$$p(b_j| \dots) \propto Unif\left(\max\left(z_{i\in A_j}\right), \min\left(z_{i\in A_{j+1}}\right)\right)$$

where A_j is the set of all z_i's that correspond to $y_i = j$.

S3 Appendix. Brief tutorial on fitting the MN-MS model with customized code

Denis Valle

January 2019

Part of the article: Ordinal regression models for zero-inflated and/or over-dispersed count data.
Authors: Denis Valle, Kok Ben Toh, Gabriel Zorello Laporta, Qing Zhao

In this tutorial, we reproduce some of the results for the simulated data described in the main text. More specifically, we focus on simulating data from a Poisson distribution with 8 non-zero slope parameters but the code can be easily modified to simulate data from other discrete distributions and/or a different number of non-zero slope parameters. We start by first describing how the simulated data were generated to then detailing how the model was fit and displaying its results.

Simulating data

We assume that there are 500 observations and 10 covariates. By creating all pairwise interactions between these 10 covariates and adding a column of 1's for the intercept, the final design matrix has $1 + 10 + (10 \times 9/2) = 56$ columns. The function *create.data()* creates and outputs the data, requiring the user to specify the number of non-zero regression coefficients through the argument *n.nonzero.coef*.

```
rm(list=ls(all=TRUE))
set.seed(2)

#function to create data
create.data=function(n.nonzero.coef){
  n=500 #number of observations

  #create design matrix
  nbasecov=10 #number of base covariates
  tmp=matrix(runif(n*nbasecov),n,nbasecov)
  xmat=cbind(1,tmp)

  #add pairwise interaction terms
  for (i in 1:(nbasecov-1)){
    for (j in (i+1):nbasecov){
      xmat=cbind(xmat,tmp[,i]*tmp[,j])
    }
  }
  # dim(xmat)

  #standardize all covariates
  xmat1=xmat
  for (i in 2:ncol(xmat)) xmat1[,i]=(xmat[,i]-mean(xmat[,i]))/sd(xmat[,i])
  colnames(xmat1)=paste('cov',1:ncol(xmat1),sep='')

  #create regression coefficients
  betas=rep(0,ncol(xmat))
```

```
  betas[1]=1
  if (n.nonzero.coef!=0) {
    #determine which subset will be non-zero
    ind=sample(2:length(betas),size=n.nonzero.coef)
    #determine the actual value of the non-zero coefficients
    vals=sample(c(-0.5,0.5),size=n.nonzero.coef,replace=T)
    betas[ind]=vals
  }

  #create mean and response variable
  media=exp(xmat1%*%betas)
  y=rpois(n,media)
  # hist(y,main=paste(n.nonzero.coef))

  #create and output data
  dat=cbind(y,xmat1)
  setwd('U:\\modeling abundance\\appendix tutorial')
  nome=paste('simdata Poisson',c(' MS ',' betas '),n.nonzero.coef,'.csv',sep='')
  write.csv(dat,nome[1],row.names=F)

  #output true regression coefficients
  write.csv(betas,nome[2],row.names=F)
}

create.data(n.nonzero.coef=8)
```

Fitting the model

To fit this model, the main function we rely on is the function *multinom.gibbs()*. This function depends on several auxiliary functions stored in the files "multinom_MS functions.R" and "multinom_MS gibbs.R", which are provided at the end of this document.

The function *multinom.gibbs()* requires the following arguments:

- *dat*: the dataset containing the response variable, labelled as "y", and the covariates;
- *ngibbs*: the number of iterations for the Gibbs sampler;
- *covs*: the name of the covariates to be used in the regression model;
- *burnin*: number of iterations to discard as burn-in; and
- *prior.var*: prior variance for the slope coefficients.

This function outputs a list containing two matrices, where each row is a sample from the posterior distribution:

- *betas* matrix: contains the posterior distribution for the slope coefficients
- *b* matrix: contains the posterior distribution for the estimated breaks for the latent continuous variable "z". If K is the number of unique "y" values, then there will be a total of K-1 breaks

```
rm(list=ls(all=TRUE))
library('mvtnorm')
set.seed(1)

#get required functions
setwd('U:\\GIT_models\\git_MN_modelsel')
source('multinom_MS functions.R')
source('multinom_MS gibbs.R')
```

```
#get data
distrib='Poisson'
n.nonzero.coef=8
setwd('U:\\modeling abundance\\appendix tutorial')
nome=paste('simdata ',distrib,' MS ',n.nonzero.coef,'.csv',sep='')
dat=read.csv(nome,as.is=T)
covs=colnames(dat)[-c(1,2)] #remove intercept and response variable

#set Gibbs parameters and run model
ngibbs=10000 #number of Gibbs sampler iterations
prior.var=1 #prior variance for regression coefficients
res=multinom.gibbs(dat=dat,ngibbs=ngibbs,covs=covs,burnin=5000,prior.var=prior.var)

#summarize and output results
res1=apply(res$betas,2,quantile,c(0.025,0.975))
res2=t(res1)
colnames(res2)=c('CI.025','CI.975')

setwd('U:\\modeling abundance\\appendix tutorial')
nome=paste(distrib,' MN betas',n.nonzero.coef,'.csv',sep='')
write.csv(res2,nome,row.names=F)
```

Comparing the estimated and the true number of zero and non-zero regression coefficients

It is not possible to use our model to recover the exact values of the true regression coefficients because our model is based on a different model structure (e.g., likelihood and link function) than the model used to generate the data. Nevertheless, our model is able to correctly determine which coefficients are non-zero and which sign these coefficients have. More specifically, we determine that a coefficient was correctly estimated if:

- the true coefficient is greater than zero and the lower and upper bounds of the 95% credible interval (CI) are positive;
- the true coefficient is smaller than zero and the lower and upper bounds of the 95% CI are negative; or
- the true coefficient is equal to zero and the lower and upper bounds of the 95% CI encompass zero.

Our results reveal that the proposed model is able to correctly identify the 8 non-zero regression coefficients and their signs while at the same time correctly identifying the regression coefficients that are exactly equal to zero.

```
rm(list=ls(all=TRUE))

#get estimated coefficients
distrib='Poisson'
n.nonzero.coef=8
setwd('U:\\modeling abundance\\appendix tutorial')
nome=paste(distrib,' MN betas',n.nonzero.coef,'.csv',sep='')
estim=read.csv(nome,as.is=T)

#get true coefficients
nome=paste('simdata ',distrib,' betas ',n.nonzero.coef,'.csv',sep='')
true1=read.csv(nome,as.is=T)$x
true2=true1[-1] #remove intercept
```

```
#compare true and estimated coefficients
estim$true=true2

#number of correctly classified important covariates
cond=(estim$true>0 & estim$CI.025>0 & estim$CI.975>0) |
     (estim$true<0 & estim$CI.025<0 & estim$CI.975<0)
sum(cond)   #estimated result
```

```
## [1] 8
```

```
n.nonzero.coef #expected result
```

```
## [1] 8
```

```
#number of correctly classified non-important covariates
cond=(estim$true==0 & estim$CI.025 <= 0 & estim$CI.975 >=0)
sum(cond)         #estimated result
```

```
## [1] 47
```

```
56-1-n.nonzero.coef  #expected result
```

```
## [1] 47
```

Additional code

Here we provide the underlying code behind the function *multinom.gibbs()*. This code is distributed in two files: "multinom_MS functions.R" and "multinom_MS gibbs.R". These two files are also available in our public GitHub repository https://github.com/drvalle1/git_MN_modelsel.

1) File "multinom_MS gibbs.R" containing the wrapper function *multinom.gibbs()*

```
multinom.gibbs=function(dat,ngibbs,covs,burnin,prior.var){
  nobs=nrow(dat)

  tmp=sort(unique(dat$y)) #unique response values, sorted from smallest to largest
  nclass=length(tmp) #number of unique classes
  class1=1:nclass    #transformed variable (i.e., ranks)
  uni=data.frame(y=tmp,y1=class1) #mapping of unique response values to rank values

  dat1=merge(dat,uni,all=T); dim(dat); dim(dat1)
  dat2=dat1[,c('y1',covs)]

  #get initial values
  z=(dat2$y1-mean(dat2$y1))/sd(dat2$y1)
  b=class1[1:(nclass-1)]+0.1
  b=(b-mean(dat2$y1))/sd(dat2$y1)
  xmat.orig=cov=data.matrix(dat2[,covs])
  maxp=ncol(cov)

  betas=rep(0,maxp)
  indin=1:4
  indout=5:maxp
  betas=betas[indin]
  cov=cov[,indin]
```

```r
  #gibbs sampler
  store.b=matrix(NA,ngibbs,nclass-1)
  store.betas=matrix(NA,ngibbs,maxp)
  options(warn=2)
  for (i in 1:ngibbs){
    print(c(i,indin))
    # print(c(i,b))

    #to flag when a covariate is chosen multiple times
    tmp=table(indin)
    if (sum(tmp>1)>0) break;

    z=sample.z(y=dat2$y1,cov=cov,betas=betas,b=b,class1=class1,nobs=nobs,nclass=nclass)
    b=sample.b(z=z,y=dat2$y1,nclass=nclass,class1=class1)

    tmp=samp.move(indin,indout,maxp,z,xmat.orig,prior.var)
    indin=tmp$indin
    indout=tmp$indout
    cov=tmp$cov

    betas=sample.betas(z=z,cov=cov,prior.var=prior.var)

    #store results
    store.b[i,]=b

    tmp=rep(0,maxp)
    tmp[indin]=betas
    store.betas[i,]=tmp
  }

  after.burn=burnin:ngibbs
  list(b=store.b[after.burn,],
       betas=store.betas[after.burn,])
}
```

2) File “multinom_MS functions.R” containing auxiliary functions written in R

```r
#generates truncated normal variates based on cumulative normal distribution
#normal truncated lo and hi
tnorm <- function(n,lo,hi,mu,sig){

  if(length(lo) == 1 & length(mu) > 1)lo <- rep(lo,length(mu))
  if(length(hi) == 1 & length(mu) > 1)hi <- rep(hi,length(mu))

  q1 <- pnorm(lo,mu,sig) #cumulative distribution
  q2 <- pnorm(hi,mu,sig) #cumulative distribution

  z <- runif(n,q1,q2)
  z <- qnorm(z,mu,sig)

  #qnorm can give some imprecise results
  cond=z<lo;    z[cond] = lo[cond]
  cond=z==-Inf; z[cond] = lo[cond]
```

```r
  cond=z>hi;    z[cond] = hi[cond]
  cond=z==Inf;  z[cond] = hi[cond]
  z
}
#-----------------------------------------------------------------------------------------------
sample.z=function(y,cov,betas,b,class1,nobs,nclass){
  z=rep(NA,nobs)
  media=cov%*%betas

  #------------------------------
  #get z
  cond=y==class1[1]
  z[cond]=tnorm(sum(cond),lo=-100,hi=b[1],mu=media[cond],sig=1)

  for (i in 2:(nclass-1)){
    cond=y==class1[i]
    z[cond]=tnorm(sum(cond),lo=b[i-1],hi=b[i],mu=media[cond],sig=1)
  }

  cond=y==class1[nclass]
  z[cond]=tnorm(sum(cond),lo=b[nclass-1],hi=100,mu=media[cond],sig=1)
  z
}
#-----------------------------------------------------------------------------------------------
sample.betas=function(z,cov,prior.var){
  tmp=dim(cov)
  if (is.null(tmp)){
    p=1
    prec=matrix(t(cov)%*%cov+(1/prior.var),1,1)
  }
  if (!is.null(tmp)){
    p=ncol(cov)
    prec=t(cov)%*%cov+diag(1/prior.var,p)
  }
  var1=solve(prec)
  pmedia=var1%*%t(cov)%*%z
  t(rmvnorm(1,pmedia,var1))
}
#-----------------------------------------------------------------------------------------------
sample.b=function(z,y,nclass,class1){
  b=rep(NA,nclass-1)
  for (i in 2:nclass){
    cond1=y==class1[i-1]
    cond2=y==class1[i]
    lo=max(z[cond1])
    hi=min(z[cond2]);
    # if (lo==hi) hi=hi+0.001
    b[i-1]=runif(1,lo,hi)
  }
  b
}
#------------------------------
log.marg.likel=function(cov,z,prior.var){
```

```
  tmp=dim(cov)
  if (is.null(tmp)){
    p=1
    prec=matrix(t(cov)%*%cov+(1/prior.var),1,1)
  }
  if (!is.null(tmp)){
    p=ncol(cov)
    prec=t(cov)%*%cov+diag(1/prior.var,p)
  }
  var1=solve(prec)
  mu=var1%*%t(cov)%*%z

  -(1/2)*(-t(mu)%*%prec%*%mu+t(z)%*%z+p*log(prior.var)-determinant(var1)$modulus[1])
}
#-------------------------------------
samp.move=function(indin,indout,maxp,z,xmat.orig,prior.var){
  indin.old=indin
  p=length(indin.old)
  rand1=runif(1)
  p0=1
  if (p == 1) {
    if (rand1 < 1/2) indin.new=swap(indin,indout)
    if (rand1 > 1/2) indin.new=birth(indin,indout)
    p0=2/3 #death prob 2 -> 1 is (1/3) and birth prob 1 -> 2 (or swap prob 1 -> 1) is 1/2.
  }
  if (p == maxp) {
      indin.new=death(indin)
      p0=1/3 #birth prob T-1 -> T is (1/3) and death prob T -> T-1 is 1
  }
  if (1 < p & p < maxp) {
    if (rand1 < 1/3) {
      indin.new=birth(indin,indout)
      if (p==maxp-1) p0=3 #death prob from T -> T-1 is 1 and birth prob from T-1 -> T is (1/3)
    }
    if (1/3 < rand1 & rand1 < 2/3) {
      indin.new=death(indin)
      if (p==2) p0=3/2 #birth prob from 1 -> 2 is 1/2 and death prob from 2 -> 1 is 1/3
    }
    if (2/3 < rand1) indin.new=swap(indin,indout)
  }
  pold=log.marg.likel(cov=xmat.orig[,indin.old],z=z,prior.var=prior.var)
  pnew=log.marg.likel(cov=xmat.orig[,indin.new],z=z,prior.var=prior.var)+log(p0)
  prob=exp(pnew-pold)
  rand2=runif(1)

  seq1=1:maxp
  k=which(!seq1%in%indin.new)
  indout.new=seq1[k]
  if (rand2<prob) return(list(cov=xmat.orig[,indin.new],indin=indin.new,indout=indout.new))
  return(list(cov=xmat.orig[,indin.old],indin=indin.old,indout=indout))
}
#-------------------------------------------
death=function(indinz){
```

```
  k=sample(1:length(indinz),size=1)
  indinz[-k]
}
#-----------------------------------------------------------------------------------------
swap=function(indinz,indoutz){
  n=length(indinz)
  if (n==1) tmp=numeric()
  if (n >1) {
    k=sample(1:n,size=1)
    tmp=indinz[-k]
  }

  n=length(indoutz)
  if (n==1) include=indoutz
  if (n> 1) include=sample(indoutz,size=1)
  sort(c(tmp,include))
}
#-----------------------------------------------------------------------------------------
birth=function(indinz,indoutz){
  n=length(indoutz)
  if (n==1) k=indoutz
  if (n>1 ) k=sample(indoutz,size=1)
  sort(c(indinz,k))
}
```
